# Altered adipocyte differentiation and unbalanced autophagy in type 2 Familial Partial Lipodystrophy: an in vitro and in vivo study of adipose tissue browning

**DOI:** 10.1038/s12276-019-0289-0

**Published:** 2019-08-02

**Authors:** Camilla Pellegrini, Marta Columbaro, Elisa Schena, Sabino Prencipe, Davide Andrenacci, Patricia Iozzo, Maria Angela Guzzardi, Cristina Capanni, Elisabetta Mattioli, Manuela Loi, David Araujo-Vilar, Stefano Squarzoni, Saverio Cinti, Paolo Morselli, Assuero Giorgetti, Laura Zanotti, Alessandra Gambineri, Giovanna Lattanzi

**Affiliations:** 1 CNR - National Research Council of Italy, Institute of Molecular Genetics “Luigi Luca Cavalli-Sforza”, Unit of Bologna, Bologna, Italy; 2 IRCCS, Istituto Ortopedico Rizzoli, Bologna, Italy; 3 CNR - National Research Council of Italy, Institute of Clinical Physiology, Pisa, Italy; 4 Department of Medicine, CIMUS Biomedical Research Institute, University of Santiago de Compostela, Santiago de Compostela, Spain; 5 Department of Experimental and Clinical Medicine, University of Ancona (UniversitàPolitecnicadelle Marche), Ancona, Italy; 6 Center of Obesity of University of Ancona, Ancona, Italy; 7 Plastic Surgery Unit, Department of Specialised, Experimental, and Diagnostic Medicine, Alma Mater Studiorum University of Bologna, S Orsola-Malpighi Hospital, Bologna, Italy; 8 Fondazione Toscana Gabriele Monasterio(FTGM), Pisa, Italy; 9 Endocrinology Unit, Department of Medical and Surgical Sciences, Alma Mater Studiorum University of Bologna, S Orsola-Malpighi Hospital, Bologna, Italy

**Keywords:** Nuclear envelope, Metabolic syndrome, Macroautophagy, Translational research, Mechanisms of disease

## Abstract

Type-2 Familial Partial Lipodystrophy is caused by *LMNA* mutations. Patients gradually lose subcutaneous fat from the limbs, while they accumulate adipose tissue in the face and neck. Several studies have demonstrated that autophagy is involved in the regulation of adipocyte differentiation and the maintenance of the balance between white and brown adipose tissue. We identified deregulation of autophagy in laminopathic preadipocytes before induction of differentiation. Moreover, in differentiating white adipocyte precursors, we observed impairment of large lipid droplet formation, altered regulation of adipose tissue genes, and expression of the brown adipose tissue marker UCP1. Conversely, in lipodystrophic brown adipocyte precursors induced to differentiate, we noticed activation of autophagy, formation of enlarged lipid droplets typical of white adipocytes, and dysregulation of brown adipose tissue genes. In agreement with these in vitro results indicating conversion of FPLD2 brown preadipocytes toward the white lineage, adipose tissue from FPLD2 patient neck, an area of brown adipogenesis, showed a white phenotype reminiscent of its brown origin. Moreover, in vivo morpho-functional evaluation of fat depots in the neck area of three FPLD2 patients by PET/CT analysis with cold stimulation showed the absence of brown adipose tissue activity. These findings highlight a new pathogenetic mechanism leading to improper fat distribution in lamin A-linked lipodystrophies and show that both impaired white adipocyte turnover and failure of adipose tissue browning contribute to disease.

## Fat tissue disorders: Dysfunctional fat cell differentiation

An abnormal distribution of fatty tissues associated with certain tissue disorders is driven by disrupted fat cell differentiation. Type 2 familial partial lipodystrophy (FPLD2) is a genetic condition that results in fat being lost from the limbs and accumulating in the face and neck. Giovanna Lattanzi at the National Research Council of Italy in Bologna and co-workers found that fat cell (adipocyte) precursors did not clearly differentiate into either of the two main fatty tissue types, brown or white, in FPLD2 patients. White adipocyte precursors exhibited impaired lipid formation and abnormal levels of brown tissue markers. Conversely, brown adipocyte precursors showed high lipid levels and increased autophagy, a natural process involving degradation and recycling of cellular components. The neck is normally where brown fat accumulates, but FPLD2 patients had adipocytes there displaying white fat characteristics.

## Introduction

Lipodystrophic syndromes are rare heterogeneous genetic diseases associated with *LMNA*, *PPARG*, *BSCL2*, or other gene mutations or acquired diseases induced by the use of some human immunodeficiency virus (HIV) protease inhibitors^1,2^. These diseases are characterized by generalized or partial fat dystrophy associated with metabolic complications comprising severe insulin resistance, diabetes, dyslipidemia, and non-alcoholic fatty liver disease^2–4^.

The phenotype of nuclear envelope-linked lipodystrophies ranges from the typical Familial Partial Lipodystrophy of the Dunnigan type (also called type 2 Familial Partial Lipodystrophy, FPLD2) due to heterozygous mutations of the *LMNA* gene encoding lamin A/C to complex diseases that can combine lipodystrophy, metabolic complications, bone resorption with osteoporosis and osteolysis, and signs of accelerated aging^3,5,6^.

FPLD2 (OMIM - # 151660) is a rare form of genetic lipodystrophy with onset around puberty. The disease is caused by the accumulation of mutated forms of lamin A precursor (prelamin A) in patient cells^5,7^, leading to altered transcription factor import^5,6,8^. A hotspot for FPLD2 is codon482 of the gene (p.R482W/Q or L mutation), although mutations can be found even in the protein N-terminal domain. FPLD2 clinical features include anomalous fat distribution (loss of peripheral subcutaneous fat and abnormal accumulation of fat in the neck and face), severe metabolic alterations with insulin resistance, generalized dyslipidemia, and early cardiovascular complications^3^.

Recent advances in the pathogenesis of different types of lipodystrophy generally pointed to primary adipocyte alterations leading to impaired adipogenesis and/or deregulation of the adipocyte lipid droplets^2^. However, the precise mechanisms linking nuclear envelope abnormalities to lipodystrophies remain largely unknown^3^. Physiological mechanisms that regulate brown adipose tissue (BAT) or white adipose tissue (WAT) distribution in the body have been recently addressed^9,10^, and major players and rules have been identified^11,12^. The adipose organ, an organ recently recognized to have anatomical and functional features, may contain brown and/or white adipocytes depending on the body district, age, and environmental stimuli^12^. Brown adipocytes are multilocular cells and contain high number of mitochondria that express uncoupling protein 1 (UCP1), a protein that shifts mitochondrial activity toward heat production. White adipocytes are typically cells with a unique enlarged vacuole that stores lipids and can undergo lipolysis when required^12^. Exposure to cold increases BAT amount and activity, especially in BAT districts, which in humans are around the major blood vessels, the supraclavicular region, and the neck^10,12^. Conversely, obesogenic conditions favor an increase of white adipocytes in the adipose organ, mostly in subcutaneous and visceral fat depots^12,13^. A major player in BAT formation is adrenergic signaling, mostly mediated by beta 3 adrenoreceptors. Indeed, BAT is highly innervated by noradrenergic fibers. As a general rule, BAT formation is mainly due to the conversion of white adipocytes. In recent years, several studies have reported that autophagy, a lysosomal degradation pathway, is involved in the regulation of lipid metabolism and in the maintenance of the balance between WAT and BAT^12,14,15^. Activation of autophagy is fundamental in WAT differentiation by repressing the proteasome-dependent degradation of PPARγ2^13^ and eliciting a reduction in mitochondrion number^16^ as well as activation of lipid droplet fusion^17^. Autophagy is upregulated in the adipose tissue of obese subjects. In contrast, chemical inhibition of autophagy or knockdown of critical macroautophagy genes (*ATG7* or *ATG5*)^17,18^ led to browning of WAT, thereby reducing lipid accumulation^15^.

Based on our morphological observations of FPLD2 tissue and cells, the latter showing the accumulation of autophagic vesicles in laminopathic preadipocytes derived from neck adipose tissue, we wondered whether an autophagic alteration could occur in FPLD2 and influence adipocyte determination and differentiation. Studies performed by other authors in human mesenchymal stem cells accumulating prelamin A showed altered activity of the transcription factor Oct-1, which affected the expression of genes in the mammalian target of rapamycin (mTOR) pathway^19^, the main pathway of autophagic signaling. Our experimental approach was based on the use of brown preadipocytes from FPLD2 patient neck or white adipocyte precursors induced to accumulate prelamin A. In laminopathic adipocyte precursors, we could determine early activation and block of autophagy. These events were associated with impaired differentiation of WAT precursors and differentiation of brown preadipocytes toward the WAT phenotype.

Thus, we suspected an in vivo alteration of BAT activity in FPLD2 patients. A previous study demonstrated UCP1 positivity of adipose tissue derived from the neck area of FPLD2 patients^20^, supporting the hypothesis that fat from that district, although showing a WAT appearance, could be of brown origin. Here we confirmed that FPLD2 adipose tissue from the neck is composed of an intermediate brown–white fat that expresses molecular markers of BAT^10^. Moreover, by in vivo imaging of fat distribution in FPLD2 patients, we showed a functional failure of BAT. The recognized method to estimate BAT activity in vivo is the ^18^F-2-fluoro-2-deoxy-D-glucose (^18^F-FDG) positron-emission tomographic and computed tomographic (PET/CT) scan with cold stimulation^9,21^. Previous studies have demonstrated that in normal subjects, cold stimuli induce browning in fat depots of the face, neck, and supraclavicular regions^9,22,23^. The PET/CT analysis with cold stimulation of three women affected by FPLD2 demonstrated the absence of active BAT depots in all examined subjects, consistent with the observed dysregulation of adipocyte differentiation. As a whole, the biochemical, morphological, and in vivo studies reported here are consistent with a pathogenetic hypothesis that links unscheduled autophagic activation to altered BAT and WAT turnover in FPLD2.

## Materials and methods

### Cell culture and transfection

BAT specimens were obtained from perithyroid biopsies of subjects undergoing surgery for benign thyroid disorders or from FPLD2 patients carrying the classical FPLD2 mutations R482Q or R482W (female, mean age 30 years) undergoing aesthetic surgery in the neck and perithyroid area, upon written consent. These samples were used to test protein expression or to establish brown adipocyte precursor cultures. Skin biopsies obtained from healthy volunteers undergoing orthopedic surgery or FPLD2 patient biopsies obtained during routine diagnostic protocols served for the isolation of human subcutaneous fat (WAT). Individual data were handled confidentially and anonymously according to the local and European ethical rules.

All cell cultures belonged to the BioLaM biobank with approval of the Rizzoli Orthopedic Institute Ethical Committee number 0018250. Preadipocytes were seeded in flasks and maintained in Dulbecco’s modified Eagle’s medium (DMEM) supplemented with 20% fetal calf serum (FCS), penicillin, and streptomycin (growth medium).

To induce prelamin A accumulation in control cells, the non-peptidomimetic drug *N*-acetyl-*S*-farnesyl-l-cysteine methylester (AFCMe, 10 μM) that blocks prelamin A maturation^24^, thus inducing accumulation of farnesylated prelamin A, was added to culture medium of a subset of primary white preadipocytes.

Control white or brown preadipocytes at day 10 or 20 in differentiation medium were transfected with wild-type (WT) *LMNA* or R482Q*LMNA* FLAG-tagged plasmids and/or with GFP-LC3II using Amaxa Nucleofector according to manufacturer’s instructions and published protocols^25^.

To block autophagic activity, a subset of adipocytes was treated with 25 μM chloroquine^6^.

### White and brown adipogenic differentiation

To induce preadipocyte differentiation, white and brown adipocyte precursors at day 2 post confluence were shifted in induction medium composed of DMEM plus 10% FCS supplemented with 0.85 µM insulin (19278, Sigma), 0.2 nM triiodothyronine (T3) (T-2877, Sigma), 1 µM dexamethasone (265005, CalBiochem), 0.5 mM isobutylmethylxanthine (I-5879, Sigma) and 125 nM indomethacin.

For white adipogenic differentiation, after 3 days of induction, the medium was replaced by white differentiation medium (WAT medium): DMEM plus 10% FCS supplemented with 0.85 µM insulin (19278, Sigma), 0.2 nM triiodothyronine (T3) (T-2877, Sigma), 100 nM pioglitazone (E6910, Sigma), and 125 nM indomethacin. WAT medium was replaced every other day and cells were allowed to differentiate for 6 additional days.

For brown adipogenic differentiation, after 3 days of induction, the medium was replaced by brown differentiation medium (BAT medium): DMEM plus 10% FCS, 0.85 µM insulin, 20 nM T3, and 100 nM pioglitazone. Cells were maintained in BAT medium for 17 days and the medium was replaced every other day. Twelve hours before the end of the differentiation protocol, cells were treated with 10 μM forskolin (F-3917, Sigma) and 1 μM pioglitazone directly added to the differentiation medium (total days 20).

### Antibodies

Antibodies employed for western blotting analysis or immunofluorescence labeling were as follows: anti-lamin A/C, goat polyclonal (SC-6215, Santa Cruz Biotechnology); anti-prelamin A, goat polyclonal (SC-6214 Santa Cruz Biotechnology); anti-prelamin A3, rabbit polyclonal^26^; PPARγ rabbit polyclonal (Cell Signaling Technology); anti-UCP1 antibody (Santa Cruz); anti-TOM20 antibody (Millipore); anti-LC3 rabbit polyclonal (NB100-2220, Novus Biological); anti-P62/SQSTM1, guinea pig polyclonal (GP62-C, Progen Biotechnik); anti-p70S6 kinase and anti-phospho-p70S6 kinase (Thr389) (Cell Signaling Technology); anti-Erk 1/2 and anti-phospho Erk1/2 (Thr202/Tyr204) (Cell Signaling Technology); anti-actin, goat polyclonal (A1616, Santa Cruz Biotechnology); anti-GAPDH, mouse monoclonal (MAB374, Millipore).

### Immunofluorescence

Human preadipocytes grown on glass coverslips were fixed with absolute methanol at −20 °C for 7 min or 4% paraformaldehyde in phosphate-buffered saline (PBS) at room temperature for 10 min. Cells fixed in paraformaldehyde were permeabilized using 0.15% Triton X-100. After saturation of nonspecific binding with PBS containing 4% bovine srum albumin (BSA), coverslips were incubated with primary antibodies overnight at 4 °C and revealed with fluorescein isothiocyanate (FITC) or Tetramethylrhodamine (TRITC)-conjugated secondary antibodies (1 h at room temperature). Samples were mounted with an antifading reagent (Molecular Probes Life Technologies) and observed with a Nikon ECLIPSE Ni epifluorescence microscope. Images captured by NIS-Elements 4.3 software were elaborated using Photoshop CS. NIS-Elements 4.3 software was also used for quantitative analysis of fluorescent signals.

### Western blotting

Cells were lysed in buffer containing 20 mM Tris-HCl, pH 7.5, 1% SDS, 1 mM Na_3_VO_4_, 1 mM phenylmethylsulfonyl fluoride, 5% β-mercaptoethanol, and phosphatase/protease inhibitor mix (Millipore), sonicated, and centrifuged for 30 min at 4 °C. Clarified lysates were diluted in Laemmli buffer, subjected to SDS-polyacrylamide gel electrophoresis (6%, 8% or 4–20% gradient gel), and transferred to nitrocellulose membranes (Bio-Rad) overnight at 4 °C. Membranes were saturated with 4% BSA and incubated with primary antibodies for 1 h or overnight. Secondary antibodies were used at a 1:15,000 dilution for 20 min. Immunoblotted bands were revealed by the Amersham ECL detection system. Intensity measurements were performed using a Bio-Rad densitometer (GS 800) equipped with Quantity One Software.

### Electron microscopy

Cell pellets from control and FPLD2 preadipocytes were fixed with 2.5% glutaraldehyde–0.1 M cacodylate buffer pH 7.3 for 1 h at room temperature. After post-fixation with 1% osmium tetroxide (OsO4) in 0.1 M sodium cacodylate buffer for 1 h, the pellets were dehydrated in an ethanol series, infiltrated with propylene oxide and embedded in Epon resin. Ultrathin sections (60 nm thick) were stained with uranyl acetate and lead citrate (15 min each), and were observed at a 0° tilt angle with a JEOL JEM-1011 transmission electron microscope operated at 100 kV. At least 300 cells per sample were observed.

### Real-time PCR

Total RNA was extracted with TRI Reagent Solution (Ambion) and treated with TURBO DNase (Ambion). cDNAs were prepared using a High-Capacity RNA-to-cDNA Kit (Applied Biosystems) according to the manufacturer’s protocol. Gene expression was quantified by quantitative PCR using Power SYBR Green PCR master mix (Applied Biosystems) and analyzed by the StepOnePlus Real-Time PCR System (Applied Biosystems). The primers used in this study are reported in Table 1. Dissociation curve analysis was performed to determine target specificity. Transcript levels were normalized to the internal standard gene *GAPDH*.

**Table 1:** List of primers used in this study

	Forward primer	Reverse primer
GAPDH	5′-CTTAACTACCCTGTTTGTCG-3′	5′-TTGCCATGGGTGGAATCATA-3′
PRDM16	5′-TCATGGACCCCATCTACAGC-3′	5′-CCGCAGGTACTTCTCCTTCA-3′
DIO2	5′-ACTTCCTGCTGGTCTACATTGATG-3′	5′-CTTCCTGGTTCTGGTGCTTCTTC-3′
UCP1	5′-TCTCTCAGGATCGGCCTCTA-3′	5′-GCCACTCCTCCAGTCGTTAG-3′
PPARγv1	5′-AAGGCCATTTTCTCAAACGA-3′	5′-GAGAGATCCACGGAGCTGAT-3′
PPARγv2	5′-CCAGAAAGCGATTCCTTCAC-3′	5′-GAGAGATCCACGGAGCTGAT-3′
ADIPOQ	5′-CCTGGTGAGAAGGGTGAGAA-3′	5′-CACCGATGTCTCCCTTAGGA-3′
LEP	5′-TGACACCAAAACCCTCATCA-3′	5′-CCAAACCGGTGACTTTCTGT-3′
SLC4A	5′-GCCTGCCAGAAAGAGTCTGA-3′	5′-AGCCAGCACTCCAGAAACAT-3′

### In vivo study

#### Subjects

Three unmedicated women affected by FPLD2 were enrolled in the in vivo study and underwent the PET/CT study with cold stimulation. Their ages were 24, 36, and 60 years, whereas their body mass index values were 22, 25, and 26 kg/m^2^, respectively. The diagnosis of FPLD2 was genetically confirmed. In particular, two patients were heterozygous for R482Q, whereas one patient was heterozygous for R482W.

#### PET study protocol

On the day of the PET/CT session, participants were studied after a fasting period of at least 4 h. A catheter was inserted in an antecubital vein for blood withdrawal and tracer injection. Then, subjects were exposed to a 2 h cold preconditioning, including light clothing, room temperature of 21.3 ± 0.1 °C, and intermittent feet immersion (5 min in/5 min out) in cold water (4 ± 1 °C). After 2 h, subjects were transferred to the PET/CT room (21.8 ± 0.4 °C). During the PET/CT scan, a cold fan was oriented toward the patients’ feet to maintain the cold stimulus.

Blood samples were withdrawn at baseline (before the beginning of preconditioning), after 1 and 2 h from the beginning of the cold test, and at the end of the PET/CT session to measure norepinephrine and glucose levels. At the same time points, body temperature was measured and a Visual Analogue Scale assessing the degree of cold perceived (0 = no cold at all, 10 the highest cold I ever perceived) was administered to the subjects.

#### PET scanning and image processing

PET images were acquired dynamically with a PET/CT scanner (Discovery VCT, GE Healthcare, CT, USA). A bolus of 195 ± 15 MBq of the glucose analog ^18^F-2-fluoro-2-deoxy-D-glucose (^18^F-FDG) was administered and a dynamic emission scan with variable frame length (12 × 10 s, 2 × 30 s, 3 × 60 s, 2 × 120 s, 8 × 300 s) was started simultaneously to image the neck region, from the mastoid process of the temporal bone (rostrally) to the clavicular region (caudally). Next, a static total-body PET image was acquired. Small blood samples were withdrawn at 30, 40, and 50 min from the beginning of the dynamic PET acquisition to monitor glycemia.

All sinograms were corrected for deadtime, decay, and photon attenuation, and reconstructed by standard algorithms. Regions of interest (ROIs) were manually drawn in the fusion image composed of PET and CT images in all three transaxial, sagittal, and coronal planes to identify fat depots in the cheek, neck, and supraclavicular regions (density on CT between −200 and −10 Hounsfield units compatible with CT density of adipose tissue). ROIs were also drawn on the trapezius muscle. A small circular region was drawn in the clavicular region corresponding to the subclavian vessel, representing the amount of tracer available in the circulation for organ extraction. Then, regional tissue time activity curves were generated and analyzed against radioactivity in blood using the Patlak model^27^ to quantify the tissue extraction rate constant (Ki, ml/min/ml). Tissue glucose uptake (µmol/100 ml/min) was obtained by multiplying the Ki value by the mean plasma glucose concentration during PET and expressed per 100 ml of tissue volume. We additionally calculated the semiquantitative standardized uptake value (SUV) by dividing tissue radioactivity in the last PET frame by the injected dose of ^18^F-FDG (dose per kilogram). A maximal SUV of ^18^F-FDG of at least 2.0 g/ml was considered compatible with the presence of BAT.

#### Blood catecholamine measurement

To assess plasma norepinephrine levels, blood was collected in ice with glutathione [concentration]. Then, whole blood was centrifuged at 4 °C, plasma separated, and norepinephrine assessed by an high-performance liquid chromatography system (Chromsystems Instruments and Chemical GmbH) according to the manufacturer’s instructions.

#### Statistical analysis

Three biological replicates per experiment were obtained and cell cultures from three FPLD2 patients carrying the above reported mutations were used in this study. Data are expressed as the means of three or more different experiments ± SD. Statistical analyses were performed using GraphPad software or IBM SpSS Statistic, Version 20.0 for MAC. Comparisons between groups were made by Student’s *t*-test. Differences were considered significant for < 0.05 and highly significant for *p* < 0.01. A single symbol (*) indicates *p* < 0.05 and a double symbol (**) indicates < 0.01.

## Results

### Early activation and block of autophagy in laminopathic adipocyte precursors

#### Autophagy in brown adipocyte precursors

**Fig. 1:**
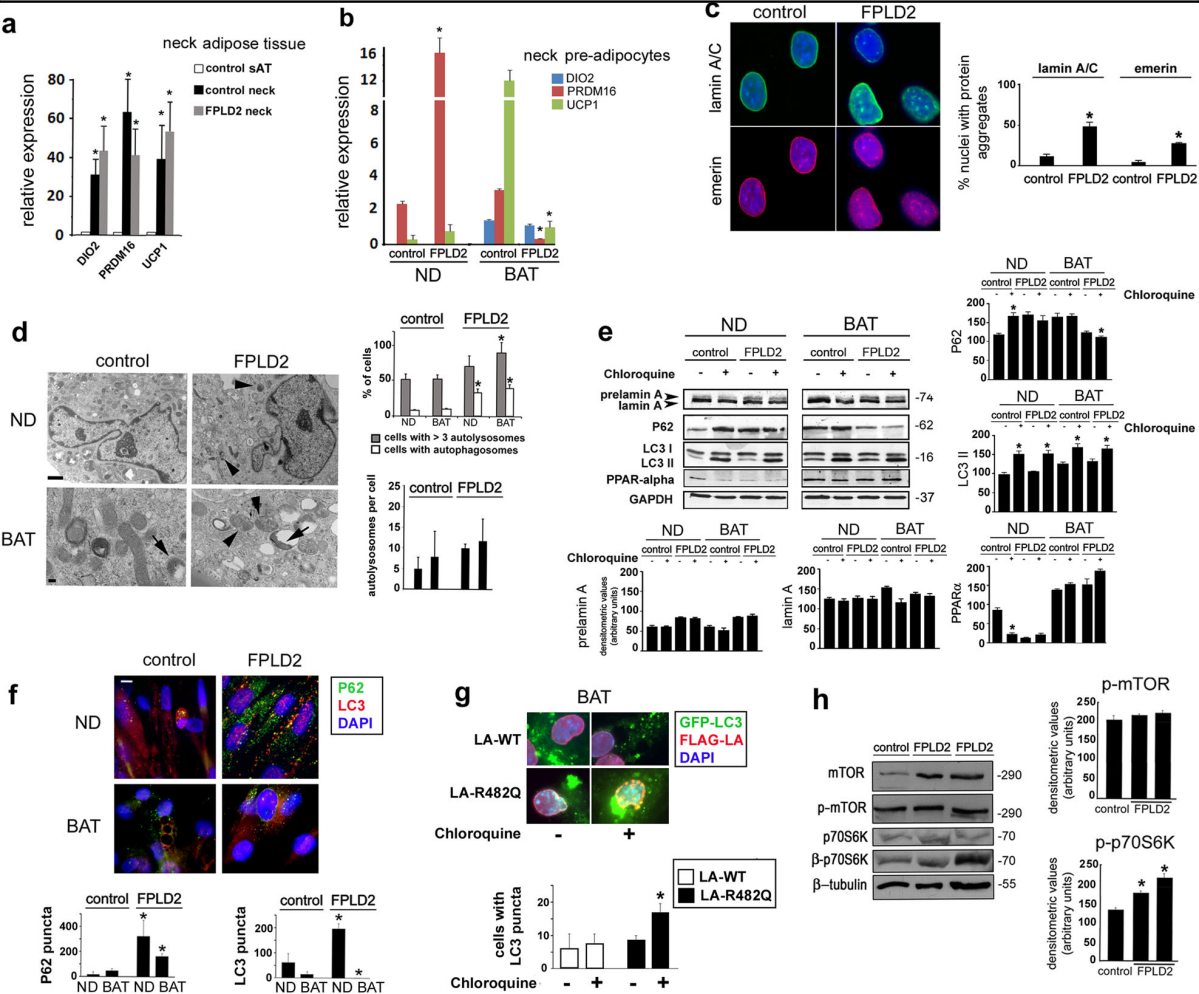
Early activation of autophagy in laminopathic brown preadipocytes. **a** Quantitative RT-PCR analysis of the BAT markers DIO2, PRDM16, and UCP1 in subcutaneous adipose tissue of control subjects (control sAT) or in adipose tissue from the neck area of controls (control neck) or FPLD2 patients (FPLD2 neck). Statistically significant differences are referred to subcutaneous adipose tissue (control sAT) values. **b** Quantitative RT-PCR analysis of the BAT markers DIO2, PRDM16, and UCP1 in control or FPLD2 neck preadipocytes either non-differentiated (ND) or after 20 days of differentiation toward the brown lineage (BAT). Statistically significant differences between values measured in control and the corresponding ND or BAT FPLD2 cells are indicated (*). **c** Control and FPLD2 neck-derived preadipocytes (FPLD2) were costained for lamin A/C (green) and emerin (red), and nuclei were counterstained with DAPI (blue). Bar, 10 μm. The percentage of nuclei with protein aggregates (lamin A/C or emerin) is reported in the graph. Data refer to 200 nuclei per sample in three independent counts. Statistically significant differences between values measured in control and FPLD2 ND cells are indicated (*). **d** Electron microscopy analysis of control (control) or FPLD2 (FPLD2) neck preadipocytes. Pictures from proliferating cells (ND) or after differentiation for 20 days (BAT), displaying mitochondria, autophagosomes (arrowheads), and autolysosomes (arrows). Bar, 1 μm in the upper panels. Bar, 100 nm in the lower panels. The percentage of cells with autophagosomes or with more than three autolysosomes and the number of autolysosomes per cell are reported in the upper graph. The number of autolysosomes per cell is reported in the lower graph. Fifty cells per sample were counted in three independent experiments. Statistically significant differences between values measured in control and the corresponding ND or BAT FPLD2 cells are indicated (*). **e** Western blotting analysis of prelamin A and the autophagic markers P62 and LC3 in control and FPLD2 preadipocytes (same samples as in **b**). PPARalpha is shown as a marker of differentiation. GAPDH bands are shown as protein loading controls. Molecular weight markers are reported in kDa. Densitometric analysis of immunoblotted prelamin A, lamin A, PPARG alpha, P62, and LC3II bands is reported in the graphs. Statistically significant differences between values measured in untreated and chloroquine-treated cells are indicated (*). **f** Immunofluorescence analysis of P62 (green) and LC3 (red) in control and FPLD2 preadipocytes (same samples as in **b**). DNA was stained with DAPI (blue). Statistical analysis of the number of P62 and LC3 puncta is reported in the graphs. Bar, 10 μm. Statistically significant differences between values measured in control and the corresponding ND or BAT FPLD2 cells are indicated (*). **g** GFP-LC3II and FLAG-LA staining in control BAT adipocytes expressing LA-WT or LA-R482Q. Statistical analysis of the number of cells with GFP-LC3II puncta is reported in the graph. Statistically significant differences between values measured in LA-WT and the corresponding LA-R482 cells are indicated (*). **h** Western blotting analysis of mTOR, p-mTOR, p70S6k, and p-p70S6K in control (control) and two different FPLD2 patient ND cell cultures (FPLD2). Tubulin bands are shown as protein-loading controls. Densitometry of phosphorylated proteins is reported in the graph. When comparing wild-type and FPLD2 samples, three biological replicates were used in each experiment as well as in qRT-PCR analyses. Statistically significant differences between values measured in control and FPLD2 cells are indicated (*)

Compared with control adipose tissue from the trunk, markers of BAT, including UCP1, type II deiodinase (DIO2), and PR domain containing 16 (PRDM16), were upregulated in control and FPLD2 adipose tissue from the neck area (Fig. 1a). Based on this result, we considered these samples to be BAT. From these tissues, we derived primary cell cultures. PRDM16 and UCP1, markers of the brown adipose lineage, were also positive in cultured preadipocytes, whereas DIO2 was only expressed in differentiated cells (Fig. 1b). Of note, PRDM16 was highly increased in FPLD2 preadipocytes, whereas very low levels of PRDM16 and UCP1 were detected in FPLD2 adipocytes with respect to reference control cells (Fig. 1b). Relative expression of BAT- and WAT-specific marker genes in white vs. brown adipocytes is shown in Supplementary Fig. S1.

FPLD2 preadipocyte nuclei showed typical laminopathic morphology^5,28,29^, with formation of honeycomb structures at the nuclear envelope and lamin A/C and emerin aggregates (Fig. 1c).

By ultrastructural analysis, we observed the accumulation of autophagic vesicles both in FPLD2 preadipocytes and in differentiated FPLD2 adipocytes but not in controls (Fig. 1d). Thus, we sought to analyze the autophagic pathway in these cells. To this end, the autophagy markers LC3 and P62 were examined^6^. P62 and the LC3II form of LC3 are known to accumulate in cells when autophagy is activated and then blocked^22^. To test autophagic flux, we used chloroquine, a known inhibitor of lysosomal activity that causes accumulation of P62 and LC3II when added to cells with active autophagy. Western blotting analysis showed accumulation of LC3II in all chloroquine-treated samples, indicating basal levels of autophagy in all cell cultures and efficient inhibition of the autophagic flux by chloroquine (Fig. 1e). In untreated control brown preadipocytes, low levels of P62 were detected by western blotting analysis, whereas P62 accumulated in FPLD2 preadipocytes (Fig. 1e). However, relative to the corresponding untreated cells, P62 was increased in chloroquine-treated control brown preadipocytes but not in FPLD2 (Fig. 1e), indicating that autophagy was an ongoing process in control cells, whereas it was impaired in FPLD2 preadipocytes. In differentiated control adipocytes, P62 accumulated and chloroquine treatment failed to increase P62 accumulation (Fig. 1e), suggesting a block of autophagy, as expected for brown adipogenesis. Conversely, low levels of P62 were detected in differentiated FPLD2 adipocytes (Fig. 1e), which suggested active autophagy, although the lack of P62 accumulation upon chloroquine treatment (Fig. 1e) could indicate an aberrant autophagic process. Consistent with these results, immunofluorescence analysis showed the presence of P62- and LC3-labeled vesicles in undifferentiated FPLD2 cells but not in controls, whereas P62 and LC3 puncta were almost undetectable in differentiated cells (Fig. 1f). Cotransfection of control adipocytes at day 20 in differentiation medium with LA-WT or LA-R482Q and GFP-LC3II was also performed to test autophagic activity. Processing of GFP-LC3II in transfected cells was tested by western blotting analysis, which showed cleavage and degradation of green fluorescent protein (GFP) in cells that did not express lamin A constructs and a sharp cleavage band corresponding to GFP in LA-WT and LA-R482Q cotransfected cells (Supplementary Fig. S2). These results suggested a reduced processing rate in LA-WT and LA-R482Q-expressing cells, because the GFP band was accumulated but not degraded^30^. GFP-LC3II puncta were detectable in LA-WT-transfected BAT adipocytes and their number was slightly but not significantly increased upon chloroquine treatment, indicating low autophagic activity (Fig. 1g). However, the number of GFP-LC3II puncta was significantly increased in LA-R482Q-expressing cells upon chloroquine inhibition of lysosomal activity, thus demonstrating active autophagy (Fig. 1g)^30^.Thus, although autophagy was detectable in control preadipocytes and was inhibited upon brown differentiation, as expected, our data indicated (1) autophagy induction and simultaneous block in undifferentiated laminopathic BAT precursors and (2) moderate autophagy activation in differentiating FPLD2 brown preadipocytes^25^.

These data were suggestive of defective autophagic signaling in FPLD2 preadipocytes and adipocytes. In agreement with this hypothesis, western blotting analysis showed unaffected levels of mTOR phosphorylation but increased phosphorylation of p70S6 kinase in FPLD2 cells (Fig. 1h). mTOR-independent activation of p70S6 kinase had been previously reported^31,32^ and will be discussed in this paper.

#### Autophagy in white adipocyte precursors

**Fig. 2:**
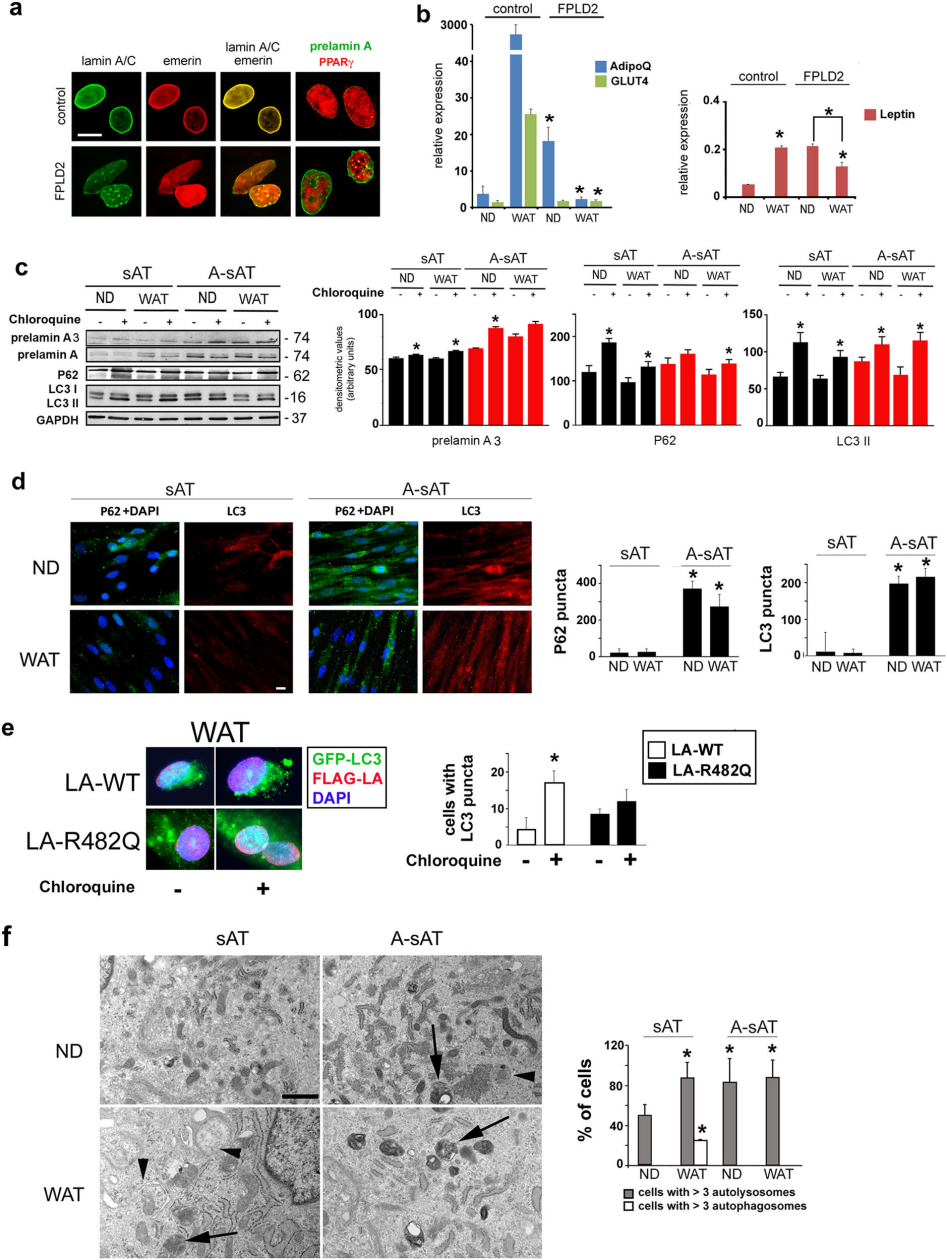
Early activation and block of autophagy in laminopathic white preadipocytes. **a** Control and FPLD2 cultured preadipocytes from subcutaneous tissue were costained for lamin A/C (green) and emerin (red) or prelamin A (green) and PPARγ (red). Bar, 10 μm. **b** Quantitative RT-PCR analysis of mRNA levels of WAT markers ADIPOq, GLUT4, and leptin in control or FPLD2 white preadipocytes, either non-differentiated (ND) or after 9 days of differentiation in WAT medium (WAT). Statistically significant differences between values measured in control and the corresponding ND or WAT FPLD2 cells are indicated (*). A significant decrease in leptin values in WAT FPLD2 vs. ND FPLD2 is also indicated (*). **c** Control cultured preadipocytes left untreated (sAT) or treated with AFCMe (A-sAT) were examined for autophagic markers. Western blotting analysis of prelamin A, P62, and LC3 (LC3I and LC3II forms) in sAT and A-sAT cells under basal conditions (ND) or differentiated in WAT medium for 9 days (WAT). GAPDH bands are shown as protein-loading controls. Molecular weight markers are reported in kDa. Densitometric analysis of immunoblotted prelamin A, P62, and LC3II bands is reported in the graphs. Statistically significant differences between values measured in untreated and the corresponding chloroquine-treated cells are indicated (*). **d** Immunofluorescence detection of P62 (green) and LC3 (red) in sAT and A-sAT cells under basal conditions (ND) or differentiated in WAT medium for 9 days (WAT). DNA was stained with DAPI (blue). Statistical analysis of the number of P62 and LC3 puncta is reported in the graphs. Statistically significant differences between values measured in sAT and the corresponding A-sAT cells are indicated (*). Bar, 10 μm. **e** GFP-LC3II and FLAG-LA staining in control WAT adipocytes expressing wild-type *LMNA* or R482Q-*LMNA*. Statistical analysis of the number of cells with GFP-LC3II puncta is reported in the graph. Fifty cells showing FLAG staining of the nuclear lamina were examined per sample. Statistically significant differences between values measured in wild-type *LMNA* and the corresponding R482-*LMNA* cells are indicated (*). **f** Electron microscopy analysis of sAT and A-sAT cells under basal conditions (ND) or after 9 days in WAT medium (WAT). Arrowheads: autophagosomes; arrows: autolysosomes. Bar, 1 μm. The percentage of cells with more than three autophagosomes/autolysosomes is reported in the graph. Fifty cells per sample were counted in three independent experiments. Three biological replicates were used in each experiment as well as in qRT-PCR analyses. Statistically significant differences between values measured in sAT and the corresponding A-sAT cells are indicated (*)

Then, we sought to analyze autophagy in WAT-derived pre-adipocytes. FPLD2 WAT-derived preadipocytes showed typical laminopathic nuclear morphology, lamin A/C and emerin aggregates, and prelamin A accumulation (Fig. 2a). Markers of WAT, including adiponectin (*ADIPOq* gene), GLUT4, and leptin, were analyzed in control and FPLD2 cells (Fig. 2b). Adiponectin and GLUT4 mRNA were expressed in all examined samples, with an increased level of adiponectin mRNA in FPLD2 preadipocytes (Fig. 2b). Notably, both *ADIPOq* and *GLUT4* mRNA expression was increased upon differentiation of control but not FPLD2 cells (Fig. 2b). Leptin expression was almost undetectable in controls, although a slightly increased level was determined in FPLD2 preadipocytes (Fig. 2b). However, the expression of leptin mRNA was increased in differentiated control cells, whereas it was decreased in FPLD2 adipocytes relative to the corresponding undifferentiated cells (Fig. 2b). Relative expression of brown and white adipocyte-specific marker genes in a comparison of in vitro differentiated white vs. brown adipocytes in parallel is shown in Supplementary Fig. S1.

As we failed to obtain a sufficient number of cells from FPLD2 WAT biopsies, we decided to mimic the condition of FPLD2 WAT-derived preadipocytes that accumulate prelamin A^5,7,33–35^. To this end, a subset of control white preadipocytes from subcutaneous tissue (here referred to as sAT cells) was treated with AFCMe. Thereafter, we refer to AFCMe-treated preadipocytes as A-sAT cells.

In undifferentiated and differentiated sAT cells, P62 and LC3II levels were increased upon chloroquine treatment (Fig. 2c), indicating active autophagy^36,37^. These results were expected, as autophagic flux has been associated with white adipogenesis^15^. However, in chloroquine-treated undifferentiated A-sAT cells, LC3II levels, but not P62 levels, were increased (Fig. 2c). These results suggested autophagic activation and block^36^. However, P62 levels were increased in chloroquine-treated A-sAT cells, indicating some induction of the autophagic process in differentiated laminopathic cells (Fig. 2c). Nevertheless, although P62-LC3-labeled autophagic vesicles were hardly detectable in sAT cells (Fig. 2d), as expected during active autophagy, all A-sAT cells showed a high number of autophagic vesicles labeled by both markers (Fig. 2d), suggestive of autophagic flux activation and block. The study of autophagic activity performed by cotransfection of wild-type or R482Q *LMNA* and GFP-LC3II plasmids in control WAT adipocytes showed accumulation of GFP-LC3II puncta in chloroquine-treated cells expressing wild-type *LMNA* but not in chloroquine-treated cells expressing R482Q *LMNA* (Fig. 2e).This result indicated inhibition of autophagic activity in cells expressing the FPLD2 *LMNA* mutation. Furthermore, ultrastructural analysis showed that the percentage of cells with more than three autolysosomes was increased in A-sAT cells under any experimental condition (Fig. 2f). This quantitative analysis indicated a block of autophagic fluxin laminopathic WAT preadipocytes^36^.

To start addressing the mechanism that elicits early autophagic activation and block in laminopathic WAT preadipocytes, we evaluated the mTOR pathway in s-AT cells expressing the FPLD2-linked R482Q *LMNA*. The P62 increase observed in differentiating mutant cells with respect to cells expressing wild-type lamin A supported the notion that autophagic activation and block occurs in laminopathic white adipocyte precursors (Supplementary Fig. S3,a). Notably, R482W *LMNA*^38^ expression induced phosphorylation of p70S6 kinase (Supplementary Fig. S3,b and c), as also observed in FPLD2 BAT precursors (Fig. 1h). Moreover, in R482W *LMNA* mutant cells, phosphorylation of Erk 1/2 (Thr389) was increased relative to WT *LMNA*-expressing cells (Supplementary Fig. S3,c). Activation of Erk 1/2 has also been detected in models of muscular laminopathies^39,40^. Notably, p70S6 kinase phosphorylation can also occur downstream of Erk 1/2 activation^41^. Overall, these data suggest that the normal autophagic process is impaired in undifferentiated laminopathic cells, possibly due to dysregulation of p70S6 kinase.

### Altered adipogenic differentiation in laminopathic cells

#### Differentiation of brown adipocyte precursors

**Fig. 3:**
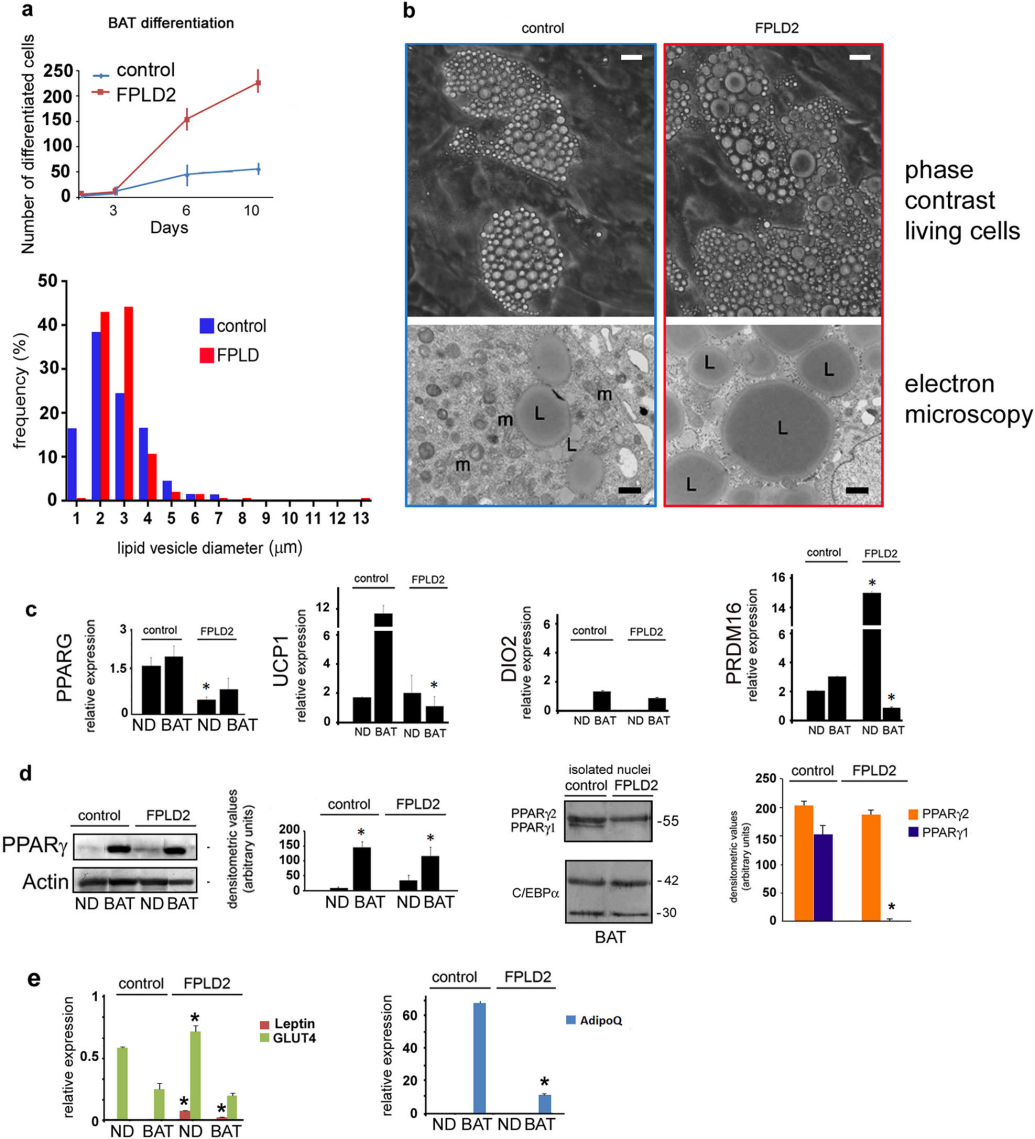
Altered differentiation of laminopathic BAT precursors. **a** Differentiation rate of control and FPLD2 BAT precursors is shown in the upper graph. The number of cells with lipid droplets observed by phase-contrast microscopy is reported. Lipid droplet diameter frequency in control and FPLD2 BAT adipocytes at day 9 of differentiation is shown in the lower graph. Data are the percentage of total counted vesicles (300 per sample in three different samples). **b** Representative images of cultured control and FPLD2 living cells at day 9 during BAT differentiation (phase-contrast living cells) and electron microscopy images of the same cell culture (electron microscopy). L, lipid droplet; m, mitochondrion. Bars, 10 μm for phase-contrast pictures, 1 μm for electron microscopy. **c** Quantification of *PPARG*, *UCP1*, *DIO2*, and *PRDM16* expression by qRT-PCR in control and FPLD2 cells under basal conditions (ND) or after 20 days of differentiation toward the brown lineage (BAT). Statistically significant differences between control and the corresponding FPLD2 cells are indicated (*). **d** Western blotting and densitometric analysis of PPARγ in control and FPLD2 adipocyte precursors under basal conditions (ND) or after 20 days of differentiation (BAT). The actin band is shown as a loading control. Western blotting and densitometric analysis of PPARγ isoforms in isolated control and FPLD2 adipocyte (BAT) nuclei is shown on the right. C-EBPα bands were stained as loading controls, indicating comparable amounts of adipocytes. Molecular weight markers are reported in kDa. **e** Quantitative RT-PCR analysis of WAT markers leptin, *GLUT4*, and *ADIPOq* in control or FPLD2 brown adipocyte precursors. Three biological replicates were used in each experiment as well as in qRT-PCR analyses. Data are means of three independent experiments ± SD. Statistically significant differences (*p* < 0.05) between control and corresponding FPLD2 samples are indicated (*)

Then, we tested the differentiation potential of laminopathic adipocyte precursors. The differentiation rate of FPLD2 neck preadipocytes induced toward the brown lineage was significantly higher than that observed in control cultures, as determined by the number of cells forming lipid droplets (Fig. 3a). However, quantitative analysis in living cells and ultrastructural analysis showed the formation of enlarged lipid droplets in differentiating FPLD2 preadipocytes (Fig. 3b). Furthermore, we observed reduced expression of *PPARG*, but not UCP1, in cycling FPLD2 preadipocytes and reduced UCP1 levels in differentiated FPLD2 adipocytes (Fig. 3c). In the same FPLD2 cell cultures, although *DIO2* expression level was comparable to controls, *PRDM16* was increased in preadipocytes and reduced in differentiated adipocytes (Fig. 3c). Notably, at the protein level, PPARγ was not apparently affected (Fig. 3d). This discrepancy was probably due to the higher number of differentiated adipocytes obtained in FPLD2 cell cultures. Notably, PPARγ1 was undetectable in FPLD2 neck preadipocytes (Fig. 3d). To support data showing the conversion of brown preadipocytes toward the white lineage in FPLD2, we tested the expression of WAT markers. Relative expression of leptin and GLUT4 mRNA was slightly increased in FPLD2 preadipocytes and adipocytes with respect to control brown adipocytes, whereas adiponectin expression was decreased (Fig. 3e).

#### Differentiation of white adipocyte precursors

**Fig. 4:**
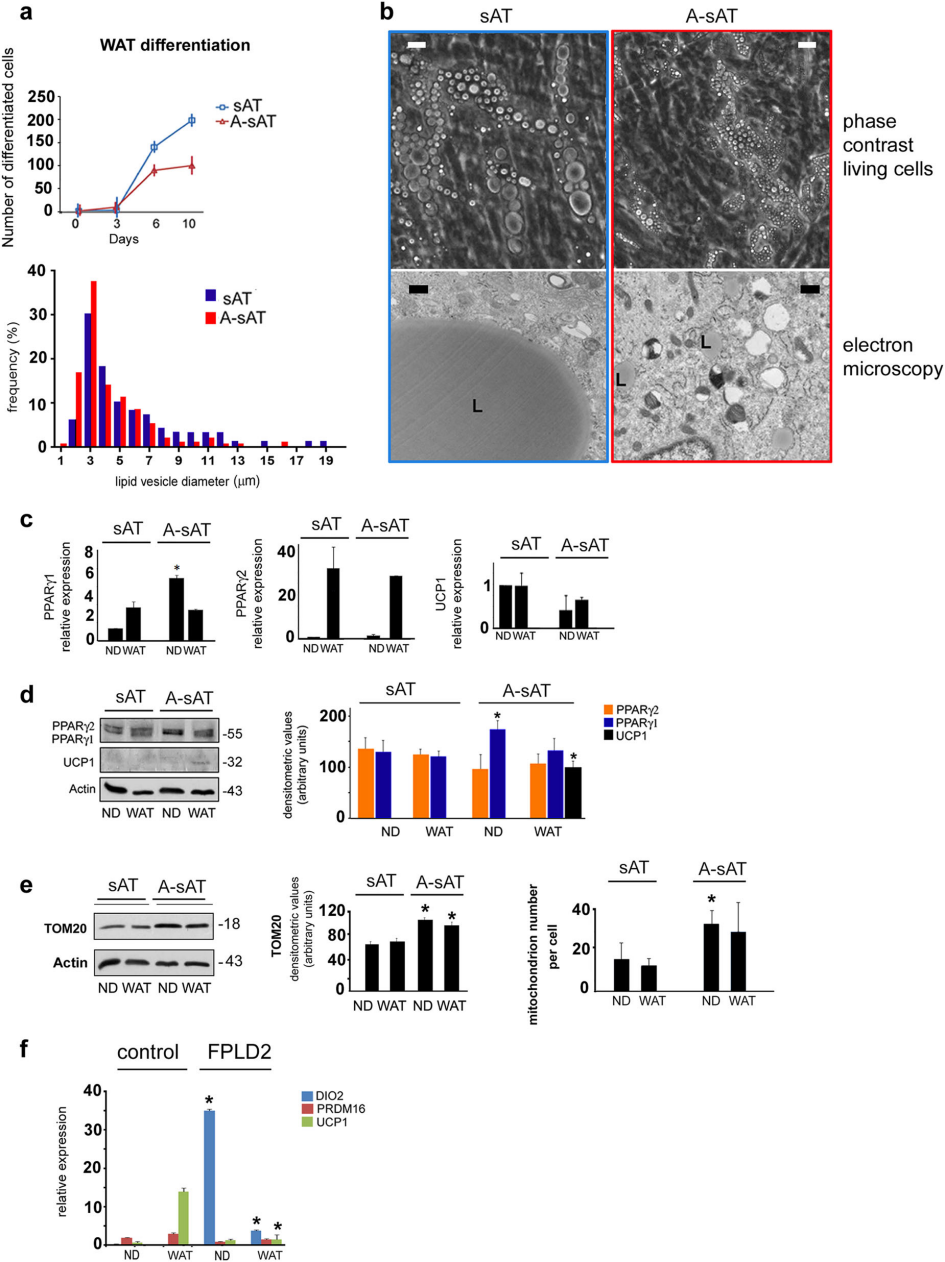
Altered differentiation of laminopathic WAT precursors. **a** Differentiation rate of control (sAT) and laminopathic WAT precursors (A-sAT) is shown in the upper graph. Lipid droplet diameter frequency in sAT and A-sAT adipocytes at day 9 of differentiation. Data are percentage of total counted vesicles (300 per sample in three different samples). **b** Representative images of cultured sAT and A-sAT living cells at day 9 in WAT medium (phase-contrast living cells) and electron microscopy images are shown (electron microscopy). L, lipid droplets. Bars, 10 μm for phase-contrast pictures, 1 μm for electron microscopy. **c** Quantification of PPARγ1, PPARγ2, and UCP1 mRNA expression by qRT-PCR in sAT and A-sAT cells under basal conditions (ND) or exposed to WAT differentiation medium for 9 days (WAT). **d** Western blotting, and densitometric analysis ofPPARγ1, PPARγ2 and UCP1 in sAT and A-sAT cells under basal conditions (ND) or exposed to WAT differentiation medium for 9 days (WAT). The actin band is shown as a protein loading control. **e** Western blotting and densitometric analysis of TOM20 in sAT and A-sAT cells under basal conditions (ND) or exposed to WAT differentiation medium for 9 days (WAT). The actin band is shown as a protein loading control. The number of mitochondria per cell in the corresponding samples as measured by electron microscopy analysis is reported in the right graph. **f** Quantitative RT-PCR analysis of BAT markers *DIO2*, *PRDM16*, and *UCP1* in control or FPLD2 white adipocyte precursors. Three biological replicates were used in each experiment as well as in qRT-PCR analyses. Data are means of three independent experiments ± SD. Molecular weight markers in **d** and **e** are reported in kDa. Statistically significant differences (*p* < 0.05) between control and corresponding FPLD2 samples are indicated by an asterisk (*)

The data reported in Fig. 4 provide insights into the differentiation potential of laminopathic white preadipocytes. A reduced differentiation rate was measured in A-sAT cells (Fig. 4a). Quantitative analysis of lipid droplet diameter in living cells (Fig. 4a) and ultrastructural analysis (Fig. 4b) allowed us to state that dimensions of lipid droplets formed in differentiating A-sAT cells were reduced. Moreover, A-sAT cells showed dramatically increased PPARγ1 mRNA levels before the onset of differentiation, whereas levels of PPARγ2 and UCP1 mRNA were comparable to controls (Fig. 4c and d). Nevertheless, the UCP1 protein level was increased in A-sAT cells, possibly due to a posttranscriptional effect. Consistent with the observed increase of UCP1, a mitochondrion membrane component, increased levels of TOM20, a protein located at the surface of the mitochondrion outer membrane, were measured in A-sAT cells (Fig. 4e). Moreover, a higher number of mitochondria per cell was observed in laminopathic preadipocytes (Fig. 4e). As a whole, these data indicated that A-sATs cells had been directed toward a brown differentiation program. In support of this observation, we found that in FPLD2 white adipocytes, the WAT genes *ADIPOq* and *GLUT4*^42^ were dysregulated, as shown above (Fig. 1b), and the BAT gene *DIO2* was upregulated (Fig. 4f).

### Adipose tissue from the FPLD2 neck district shows an intermediate brown/white phenotype and the absence of BAT activity

**Fig. 5:**
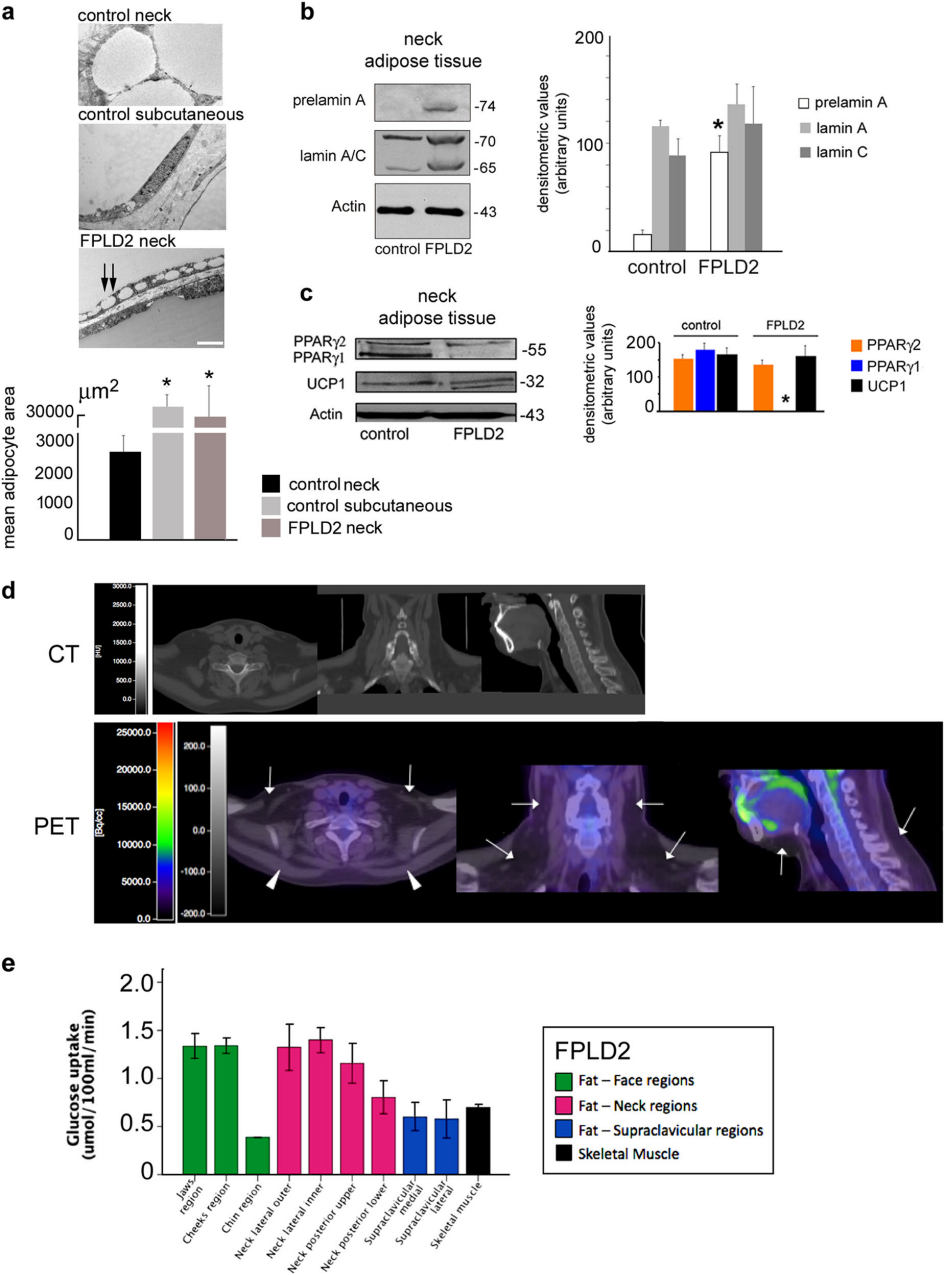
Neck FPLD2 adipose tissue shows an intermediate brown/white phenotype and absence of BAT activity. Characterization of adipose tissue isolated from the neck district of healthy donors (control neck), subcutaneous tissue from the leg of healthy donors (control subcutaneous), and FPLD2 patients neck (FPLD2 neck) is shown in **a**–**c**. **a** Electron microscopy analysis of control and FPLD2 adipose tissue. Small lipid droplets fusing to a large droplet are indicated by double arrows. Bar, 2 μm. The mean adipocyte area is reported in the graph. Statistically significant differences relative to control neck tissue are indicated (*). **b** Western blot analysis of prelamin A and lamin A/C in control and FPLD2 adipose tissue, and densitometric analysis. Statistically significant differences between control and FPLD2 tissue samples are indicated (*). **c** Western blotting analysis of PPARγ and UCP1 in control and FPLD2 adipose tissue and densitometric analysis. Statistically significant differences between control and FPLD2 tissue samples are indicated (*). In **b** and **c**: actin band is shown as a protein-loading control; molecular weight markers are reported in kDa. Cold-induced fat ^18^F-2-fluoro-2-deoxy-D-glucose (^18^F-FDG) uptake in the supraclavicular regions, face, neck, and skeletal muscle of FPLD2 patients is shown in **d**, **e**. **d** Representative CT and CT-PET images referring to one out of three examined FPLD2 patients are shown. **e** Levels of cold-induced glucose uptake (^18^F-2-fluoro-2-deoxy-D-glucose (^18^F-FDG) uptake) in fat depots of the face, neck supraclavicular regions, and trapezius muscle (skeletal muscle) of FPLD2 patients. Data are means ± SD

In agreement with the results obtained in cultured cells, FPLD2 neck adipose tissue showed an intermediate brown–white fat phenotype, as observed by electron microscopy analysis (Fig. 5). Although control adipose tissue from the neck area showed a number of small lipid droplets (diameter: 10–100 μm), FPLD2 adipose tissue appeared mostly formed by large lipid droplets (diameter: 100–200 μm or more), typical of subcutaneous WAT (Fig. 5a). As expected, we detected prelamin A (Fig. 5b) and UCP1 proteins (Fig. 5c) in adipose tissue from the FPLD2 patient neck and, interestingly, the absence of PPARγ1 (Fig. 5c), as observed in cultured preadipocytes (Fig. 3d). Thus, we concluded that the R482W lamin A mutation affects BAT features in the neck area. The in vivo study performed in FPLD2 patients strongly supported these findings, as reported below.

Cold exposure of healthy subjects induces activation of BAT in specific depots, where BAT is detectable by ^18^F-2-fluoro-2-deoxy-D-glucose (^18^F-FDG) uptake threefold higher than the other adipose tissue depots^23^. In cold-exposed healthy subjects, uptake values of ^18^F-FDG of at least 2.0 μmol/100 ml/min are considered compatible with the presence of BAT. Cold-induced fat ^18^F-FDG uptake values in several regions of the face and neck, and in the supraclavicular regions of FPLD2 patients are shown in Fig. 5d, e, together with the ^18^F-FDG uptake measured in skeletal muscle in the same conditions. The^18^F-FDG uptake in the upper and lower cheek regions was 1.34 ± 0.22 and 1.34 ± 0.14 μmol/100 ml/min, respectively, whereas the fat depot in the chin showed lower values of 0.39 ± 0.02 μmol/100 ml/min. In the neck, we identified by CT two lateral fat depots, one placed more externally (outer) and the other more internally (inner), and two dorsal fat depots, one placed in the upper and the other in the lower half of the neck. Their cold-induced ^18^F-FDG uptake values were 1.33 ± 0.42, 1.40 ± 0.23, 1.16 ± 0.36, and 0.81 ± 0.30 μmol/100 ml/min, respectively. At the supraclavicular level, medial and lateral fat depots had cold-induced ^18^F-FDG uptake of 0.60 ± 0.25 and 0.58 ± 0.28 μmol/100 ml/min, respectively. Overall, the mean value of cold-induced ^18^F-FDG uptake in all adipose depots was 1.00 ± 0.42 μmol/100 ml/min. Skeletal muscle ^18^F-FDG uptake during the cold test was 0.70 ± 0.05 μmol/100 ml/min. We also calculated the SUVs in each region. As expected, ^18^F-FDG uptake values were correlated with SUVs (*r* = 0.620, *p* < 0.002), which had a mean value of 0.46 ± 0.22 g/ml in the fat of the face region, 0.50 ± 0.15 g/ml in the neck and 0.37 ± 0.14 g/ml in the fat of the supraclavicular region. These values were incompatible with BAT, which has a described SUV of ^18^F-FDG of at least 2.0 g/ml^43,44^. During cold exposure of FPLD2 patients, skin body temperature and glycemia did not significantly change, whereas plasma norepinephrine levels increased by twofold (*p* = 0.05) and were consistent with the scoring of subjects’ own cold perception (Table 2).

**Table 2:** Clinical parameters of FPLD2 patients subjected to cold exposure with reference values

	Reference values at baseline	Baseline *T* = 0 min	Preconditioning *T* = 60 min	Start PET *T* = 120 min	Final *T* = 190 min
Body temperature (°C)	36.0–37.0	36.3 ± 0.5	36.1 ± 0.3	36.0 ± 0.4	36.2 ± 0.3
Glycemia (mg/dl)	70–110	90 ± 4	89 ± 10	96 ± 8	101 ± 13
Plasma norephinephrine (nmol/l)	< 4.0	3.7 ± 1.2	−	13.3 ± 10.3	7.1 ± 1.5*
VAS score (range)	0–3	0–3	8–9**	10**	7–8**

VAS, Visual Analogue Scale of thermal sensation and comfort

*p < 0.05 vs. baseline, **p < 0.01 vs. baseline

## Discussion

The study reported here shows that autophagy plays a role in the pathogenetic pathway of FPLD2 by altering the fate of adipogenic precursors. The first finding of our study is that early activation of autophagy occurs in undifferentiated laminopathic adipocyte precursors, but the autophagic flux does not proceed properly in those cells. How lamin A mutations and/or prelamin A accumulation interferes with autophagic pathways is in part explained by data reported by Infante, showing that an aberrant interaction between prelamin A and the transcription factor Oct-1 elicits mTOR inhibition and autophagy^19^. We previously reported sequestration of Oct-1 at the nuclear envelope in Mandibuloacral Dysplasia^6^, another *LMNA*-linked disease featuring partial lipodystrophy. Here we observed slightly reduced mTOR phosphorylation in laminopathic cells, as expected when autophagy was induced^6,45^. Nevertheless, hyperphosphorylation of p70S6 kinase, a downstream effector of mTOR signaling, was detected in FPLD2 preadipocytes. Thus, our data suggest mTOR-independent phosphorylation of p70S6 kinase in FPLD2 cells, a mechanism previously reported^31,32,46^, which deserves further investigation. Although the effect of mTOR-independent p70S6K activation on the autophagic process is still elusive, indirect evidence of a role in autophagic block is provided by a recent study performed in *Lmna*-null mice, a model of muscular laminopathies. The authors showed that partial knockout of p70S6k is sufficient to extend lifespan in knockout animals featuring autophagy impairment and premature death^47^.

Here we showed that FPLD2-linked *LMNA* mutations cause both p70S6K activation and Erk1/2 hyperphosphorylation, a defect previously associated with Mandibuloacral Dysplasia^48^ and well described in laminopathic muscle^39^ featuring autophagic impairment^49^. The different involvement of mTOR, p70S6K, and/or Erk1/2 in diverse laminopathic tissues^40^ could be relevant to the onset of disease symptoms. In this respect, the increase in phosphorylated Erk 1/2 in cells bearing FPLD2 or MADA-linked *LMNA* mutations, but not in cells bearing a mutation linked to progeria (with generalized lipoatrophy)^48^, could suggest different mechanisms that warrant investigation.

Under physiological conditions, degradation of PPARγ 2 proteases and fusion of lipid vesicles are the result of autophagic processes that occur during WAT differentiation and favor PPARγ 2 stabilization and formation of large lipid storage droplets^13^. In laminopathic precursors, we showed that autophagy is inhibited during white adipogenesis. Thus, reduced lipid vesicle fusion could be a consequence of impaired autophagy. In agreement with our results, the accumulation of autophagic markers in the absence of any lysosome inhibitor was recently reported in white preadipocytes derived from FPLD2-induced pluripotent stem cells^50^. In contrast, the autophagic process is not inhibited in differentiating BAT preadipocytes. In these cells, the formation of enlarged lipid droplets is the most likely consequence of aberrant autophagy, a process that does not take part in normal BAT development. Interestingly, although we could detect autophagic activity in brown adipocytes coexpressing GFP-LC3II and FPLD2-mutated *LMNA*, we also observed some accumulation of autophagosomes at the nuclear lamina in those cells, indicating that lamin A itself is targeted for lysosomal degradation.

In fact, lamin B^51^ and mutated lamin A^6,52,53^ may undergo autophagic degradation. As we previously observed autophagic degradation of prelamin A in MADA cells^5^, we cannot exclude the possibility that autophagic block could contribute to prelamin A accumulation in FPLD2 preadipocytes. Our present data showing autophagic impairment in FPLD2 do not allow us to determine whether A-type lamins are targeted for degradation.

As a whole, a complex network of defective signals is elicited by altered lamin A. In laminopathic WAT precursors, we observed upregulation of the γ1 isoform *PPARG* and increased protein levels. Dysregulated expression of *PPARG* before the onset of differentiation likely contributes to impaired WAT differentiation^54^, as previously shown in cells featuring an altered lamina^55^. Recently, PPARγ2 was shown to have a positive correlation with fat depot formation, whereas PPARγ1 has a negative correlation^56^. Although we did not observe any effect on PPARγ2 mRNA expression in our experimental model of laminopathic WAT, we have previously reported significantly reduced PPARγ2 mRNA levels in the thigh of FPLD2 patients, one of the most lipodystrophic districts^33^. Other depot-specific genes are also affected in laminopathic WAT precursors, supporting the hypothesis of an aberrant conversion of white preadipocytes towards the brown lineage. In fact, *ADIPOq* and *GLUT4* induction is significantly reduced in differentiating white FPLD2 adipocytes, whereas the BAT-specific gene *DIO2* is upregulated. Moreover, we observed increased levels of UCP1 protein in laminopathic WAT precursors. The expression of thermogenic genes has been associated with the formation of brite or beige adipocytes and the reduction of adipose tissue in obese animals^57^. However, recent studies have proposed a mixed phenotype of the adipose organ, which can potentially activate BAT or increase WAT depending on environmental stimuli^57^. Knockdown of the *PPARG* gene has been shown to induce both UCP1 expression and gain of a brown-like phenotype in WAT precursors^58^. However, we did not observe a transcriptional effect on the *UCP1* gene, suggesting that the observed increase in protein levels could be due to posttranslational mechanisms or could be related to the increased mitochondrion number. As a whole, both FPLD2 adipocytes and cells accumulating prelamin A showed features of brown preadipocytes during differentiation toward the white lineage. However, the data reported here showing impaired proliferation and differentiation of these cells indicate that acquisition of such an intermediate white/brown phenotype ultimately impairs WAT development. Although the ratio of adipogenic precursors in the bioptic material and in the stromal vascular fraction could also affect adipogenic differentiation in cell cultures, a reduced differentiation ability of FPLD2 adipose tissue precursors toward the white lineage is strongly suggested by the reported data.

In FPLD2 BAT precursors, reduced *PPARG* expression in differentiated cells is in agreement with previously reported transcriptional impairment^5,33,59^, although the unaffected protein levels could be related to either increased differentiation rate or stabilization of the protein. Importantly, the observed loss of PPARγ1 expression could favor pathological accumulation of fat in the neck district, as PPARγ1 downregulation has been found in WAT depots from obese subjects^60^, whereas PPARγ1 activity has been linked to catabolic pathways in adipocytes^56^. In agreement with the hypothesis of a reduced formation of BAT in the neck of FPLD2 patients, expression of the BAT marker PRDM16 was aberrantly elevated in preadipocytes and significantly decreased in FPLD2 adipocytes. In addition, UCP1 expression levels were reduced in FPLD2 neck adipocytes. These observations pave the way to a better understanding of the mechanism that specifically causes the accumulation of fat in the neck area in FPLD2 and possibly other lipodystrophies featuring the same phenotypic abnormality. Notably, the accumulation of fat in the neck area also occurs in FPLD3, a disease linked to *PPARG* mutations^1^.

In addition to molecules identified in this study, other factors, both local and systemic, might contribute to altered autophagy and/or adipocyte differentiation in FPLD2. For instance, the reported deregulation of cytokine secretion patterns most likely affects adipogenesis in Mandibuloacral dysplasia^48^, a lipodystrophic laminopathy, and further studies are needed to determine the potential pathogenetic effects of deregulated cytokines inFPLD2.

In conclusion, this study suggests that the pathogenetic mechanism leading to subcutaneous WAT loss in FPLD2 is related to early activation of autophagy in adipocyte precursors followed by autophagic flux impairment and is associated with altered regulation of PPARγ1 and UCP1. Conversely, the mechanism leading to fat accumulation in the FPLD2 neck district is related to early autophagic activation, PPARγ1 downregulation, and aberrant differentiation toward the white lineage. A puzzling result of our study is that the same *LMNA* mutations differentially affect gene expression and protein regulation in WAT or BAT. Although we cannot provide here an explanation for this effect, we suggest that differentially expressed *PPAR*γ regulators^54^ as well as differentially expressed nuclear envelope partners of lamin A^61^ may be involved.

## Supplementary information

Supplementary Figure Legends

Figure S1

Figure S2

Figure S3

## Data Availability

Data sharing was not applicable to this article because no datasets were generated or analyzed during the current study.
