# Supplementary material for: Altered adipocyte differentiation and unbalanced autophagy in type 2 Familial Partial Lipodystrophy: an in vitro and in vivo study of adipose tissue browning
Source: Exp Mol Med. 2019 Aug 2;51(8):89. doi: 10.1038/s12276-019-0289-0 (PMC6802660; doi:10.1038/s12276-019-0289-0)
Supplement: Supplementary file 1 — Supplementary Figure Legends [file 12276_2019_289_MOESM1_ESM.pdf]

**Figure S1.** Quantitative RT-PCR analysis of the expression of BAT markers DIO2, PRDM16 and UCP1, and WAT markers AdipoQ, leptin and GLUT4 in control or FPLD2 pre-adipocytes either non-differentiated (WAT nd, BAT nd) or after 20 days of differentiation towards the brown lineage (WAT diff, BAT diff). Statistically significant differences ($p<0.05$) between values measured in control and the corresponding non-differentiated or differentiated FPLD2 cells are indicated (*).

**Figure S2.** Detection of GFP-LC3 and free GFP fragments in cells expressing GFP-LC3 (GFP LC3), GFP-LC3 and FLAG-tagged wild-type *LMNA* (GFP+LA-WT) or GFP-LC3 and FLAG-tagged R482Q *LMNA* (GFP LC3 + LA-R482Q). Cellular lysates obtained from transfected HEK293 cells were subjected to 5-20% PAGE and western blot analysis using the antibodies indicated on the left (antibody). The 27 kDa band of free GFP (GFP) is obtained during active autophagy due to cleavage of GFP-LC3. Degradation of free GFP further occurs in cells undergoing the autophagic process[30]. This could be the case of cells expressing GFP-LC3 alone. Molecular weight markers (MW) are in kDa. Arrowheads indicate prelamin A (upper arrowhead) and lamin A (lower arrowhead) in transfected cells.

**Figure S3.** Activation and block of autophagy in white pre-adipocytes expressing pathogenetic R482Q *LMNA* mutant. Evaluation of autophagy in undifferentiated (ND) or differentiated (WAT) subcutaneous pre-adipocytes. Cells were either non-transfected (NT) or transfected with *LMNA*-WT or *LMNA*-R482Q constructs. (**a**) Immunofluorescence detection of FLAG-prelamin A (red) and autophagy marker P62 (green). DNA staining (DAPI) is shown in blue. Insets: 4x magnification of the indicated area. Bar, 10 μm. Representative immunofluorescence images of three independent experiments. Statistical analysis indicating the mean number of P62-labeled puncta in cells is reported in the graphs. Statistically significant differences ($p<0.05$) in mutant cells relative to the corresponding WT sample are indicated (*). (**b**) Western blotting analysis of FLAG, p70S6K and phospho-p70S6K. Actin: protein loading control. Densitometric analysis of p70S6K and phospho-p70S6K is shown in the graphs. (**c**) Western blotting analysis of FLAG, mTOR. p-mTOR, p70S6K , phospho-p70S6K, Erk 1/2 and phospho-Erk 1/2 in the corresponding samples. Tubulin: protein loading control. Molecular weight markers are reported in kDa. Densitometric analysis of immunoblotted mTOR. p-mTOR, p70S6K, p-p70S6K, Erk 1/2 and phosphor-Erk 1/2 bands is reported in the graphs. Data are means of three independent experiments ± standard deviation.
