# Supplementary figures and images for: Altered adipocyte differentiation and unbalanced autophagy in type 2 Familial Partial Lipodystrophy: an in vitro and in vivo study of adipose tissue browning
Source: Exp Mol Med. 2019 Aug 2;51(8):89. doi: 10.1038/s12276-019-0289-0 (PMC6802660; doi:10.1038/s12276-019-0289-0)

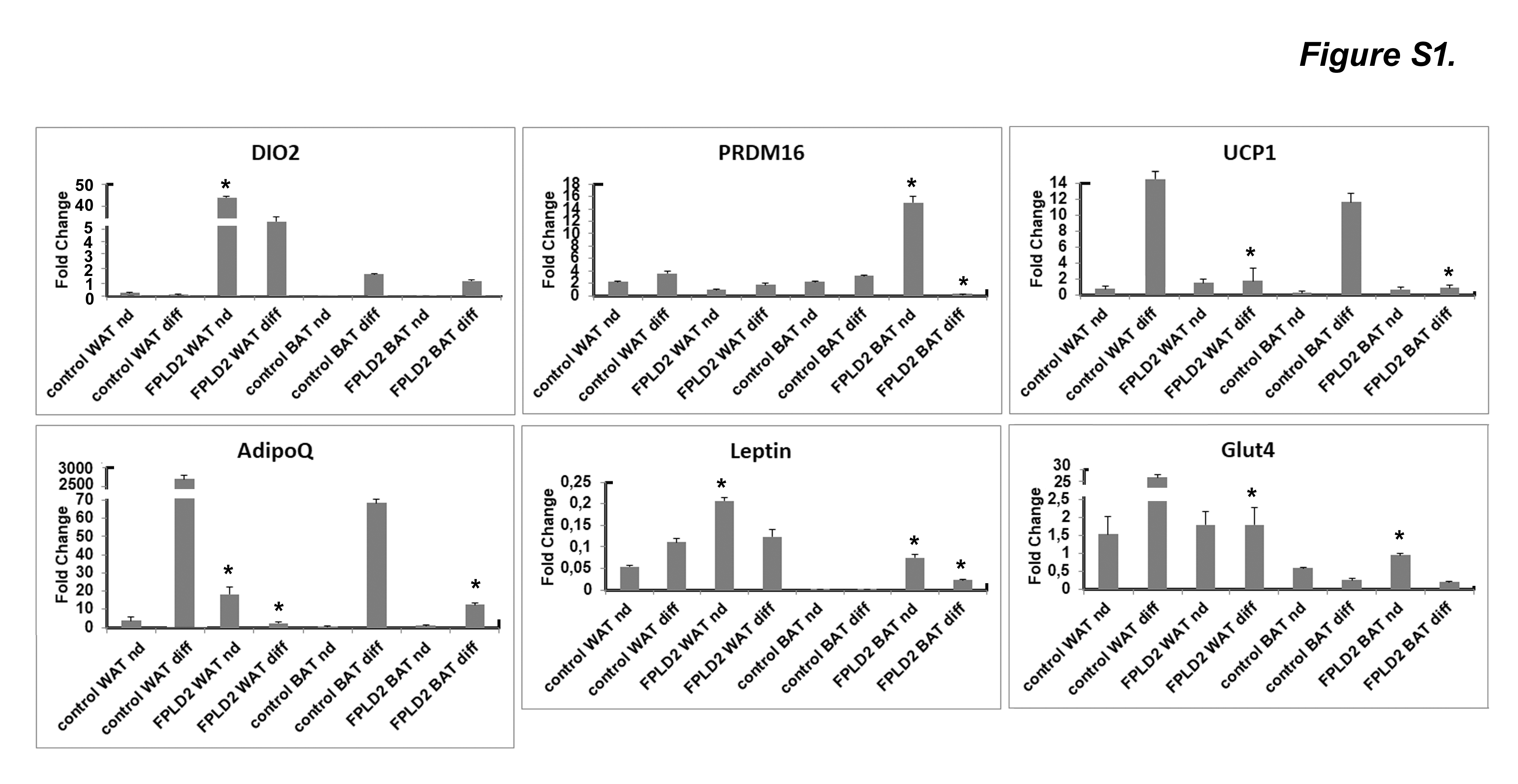

Supplement: Supplementary file 2 — Figure S1 [file 12276_2019_289_MOESM2_ESM.tif]

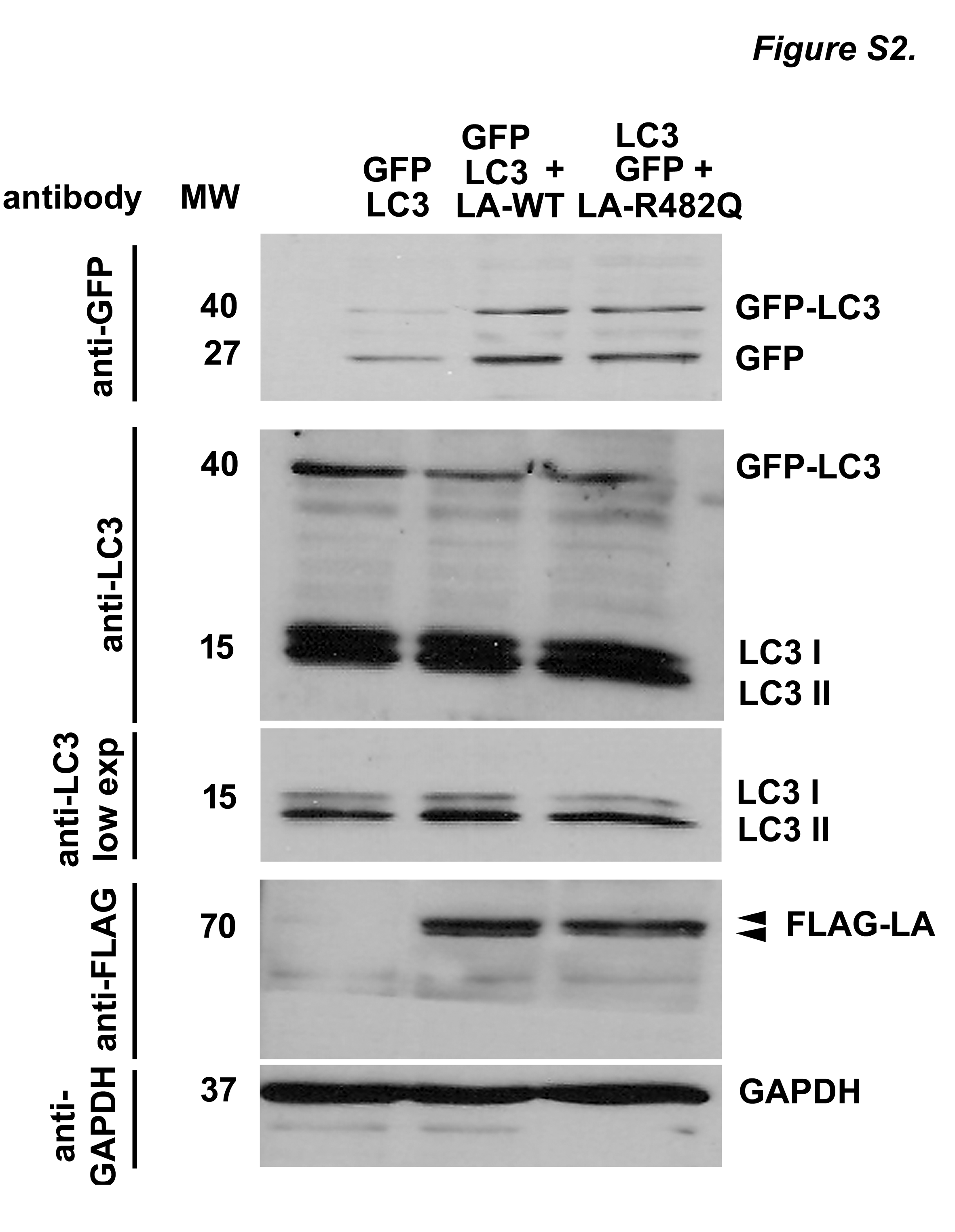

Supplement: Supplementary file 3 — Figure S2 [file 12276_2019_289_MOESM3_ESM.tif]

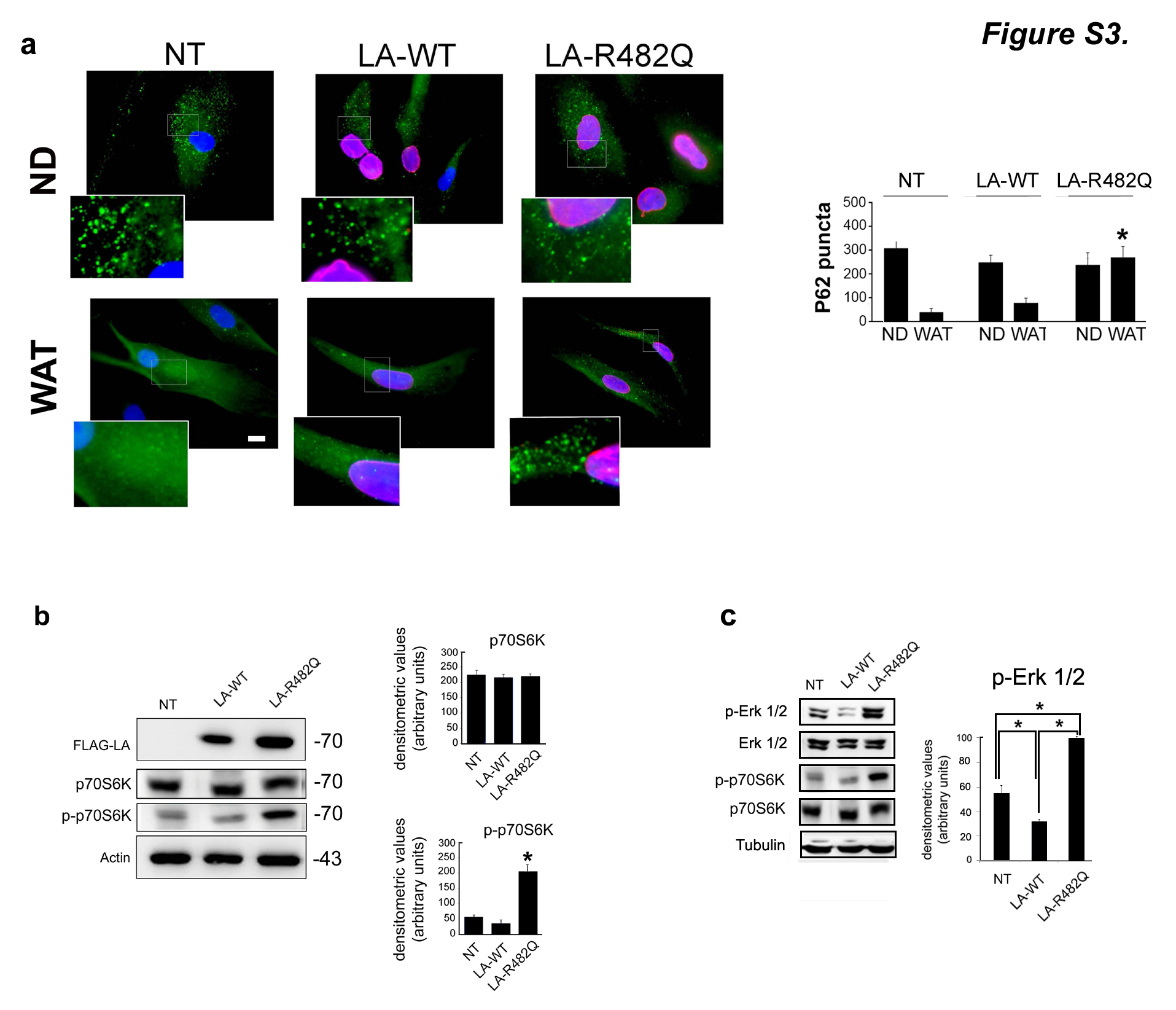

Supplement: Supplementary file 4 — Figure S3 [file 12276_2019_289_MOESM4_ESM.tif]
